# Needs assessment for behavioral parent training for ADHD in Brazil

**DOI:** 10.3389/fpsyt.2023.1191289

**Published:** 2023-07-27

**Authors:** Patricia Bado, Raquel da Costa, Camila Bernardes, Gail Tripp, Paulo Mattos, Emi Furukawa

**Affiliations:** ^1^D’Or Institute for Research and Education, Rio de Janeiro, Brazil; ^2^Department of Psychology, Pontifical Catholic University of Rio de Janeiro, Rio de Janeiro, Brazil; ^3^Okinawa Institute of Science and Technology Graduate University, Okinawa, Japan

**Keywords:** ADHD, parent training, behavioral treatment, needs assessment, Brazil

## Abstract

**Introduction:**

Attention-Deficit/Hyperactivity Disorder (ADHD) is a debilitating condition affecting children and their families worldwide. Behavioral parent training is a recommended form of empirically supported non-pharmacological intervention for young children with mild to moderate ADHD. However, access to such treatment is limited in many countries. Here we identify the treatment needs of Brazilian families with children demonstrating symptoms of ADHD, and the barriers families face in accessing behavioral treatment.

**Methods:**

A qualitative needs assessment was undertaken with parents (*n* = 23), educators (*n* = 15), and healthcare providers (*n* = 16). Semi-structured telephone interviews were conducted, and common themes were identified through inductive coding of participants’ responses.

**Results:**

Participants reported a lack of accessible behavioral treatment, and delays in accessing treatment when available. The majority of parents had not received behavioral parent training, despite it being a recommended form of treatment. Parents, educators and healthcare providers strongly endorsed a need for practical tools to manage the behavior of children with ADHD.

**Conclusion:**

Existing services might not meet the needs of children with ADHD and their families in Brazil. Easily accessed behavioral parent training programs are recommended to address the identified treatment gap for Brazilian children with ADHD and their families.

## 1. Introduction

The availability of mental health treatment for children is limited worldwide, especially in low and middle-income countries (1). This is certainly the situation in Brazil. A recent study showed that in two large cities in southern Brazil, 80% of children who need mental health interventions do not receive them (2); the rate is likely even higher in other parts of the country with fewer resources. A lack of trained professionals and infrastructure has been identified as the main reasons for such unmet needs (3, 4). These figures reflect institutional service use across the range of neurodevelopmental and psychiatric disorders. Little is known about the *individual experiences* of families using or attempting to access support for their children.

Such unmet treatment needs are exemplified by Attention Deficit and Hyperactivity Disorder (ADHD), a common neurodevelopmental disorder with a reported prevalence ranging from 2% to 7.6% (5–9). Recommended treatments include medication and psychosocial interventions. In Brazil, the most readily available treatment is pharmacotherapy; however, the published research indicates that less than 20% of children with ADHD are prescribed medication for symptom management (10). Little information is available regarding access to, and use of, empirically supported non-pharmacological interventions for ADHD (11).

Internationally, empirically supported behavioral parent training (BPT) (12, 13) alone, or in combination with other psychosocial treatments, e.g., classroom management or child skills groups (14), is recommended in the management of mild to moderate ADHD in children. A tiered approach is available and recommended in some countries; for example, group parent-training (for young children) and the provision of information about ADHD (causes and impact) together with parenting guidance and school liaison (for school-aged children), prior to more intensive individualized BPT.^1^ BPT teaches parenting strategies to encourage appropriate behavior and reduce undesired behaviors in children (15). Strategies include communicating with children in ways to increase behavioral compliance, and when and how to reward children for desired behavior to increase the likelihood of it being repeated (16). To the best of our knowledge, access to BPT in Brazil is currently very limited, with the most commonly available non-pharmacological intervention being psychoanalysis, especially in public health services (11). It is also unclear whether Brazilian parents would be motivated to take part in BPT if available.

Missing from the literature on the management of ADHD in Brazil is an understanding of the *self-reported* needs of families of children with ADHD. To address this gap, we undertook a qualitative needs assessment with the aim of identifying barriers to Brazilian families accessing psychosocial interventions, especially behavioral treatment, as well as the desired content of such support and the preferred mode of delivery. The current study focused on the first-hand experiences of adults who care for children with ADHD (17) to inform the development of an accessible BPT program, delivered online. Semi-structured interviews were conducted with parents of children with ADHD, as well as educators and healthcare providers working with these families. Interviews explored (1) experiences of seeking/providing support for children with ADHD to understand the barriers to accessing treatment and (2) information and support families currently have, need, or want. Based on their responses, common themes were identified. By incorporating the voices of parents and professionals (18), we hoped to determine whether behavioral management training would be appropriate for, and accepted by, Brazilian families and how such skills could be delivered (19, 20).

## 2. Methods

The project was reviewed and approved by the IDOR ethics committee (CAAE: 39967020.8.0000.5249). All participants were volunteers and provided written consent.

### 2.1. Participants

#### 2.1.1. Parents

Participants were parents of children previously diagnosed with ADHD by their physicians (78%) or demonstrating elevated symptoms of ADHD. The presence of comorbid conditions in the children did not exclude participation. The final sample comprised 23 parents (19 mothers, 4 fathers) with children aged 4 to 16 years, 30% girls. Most of the children received education (74%) and healthcare (78%) from the private sector. The participating families were mostly middle-class (ABEP classes B and C) (21), with the parents’ years of education ranging from 10 to 20 years.

#### 2.1.2. Educators and healthcare providers

Professionals participating in the study included 15 educators [6 education specialists (usually referred as pedagogues in Brazil), 5 teachers, 2 school principals, 2 school counselors/psychologists; 6 working in private, 6 in public and 3 in both settings], and 16 healthcare providers (5 psychiatrists, 5 psychologists, 3 speech therapists and 3 pediatricians, 7 working in private, 3 in public and 6 in both settings).

### 2.2. Procedures

Participating parents were recruited through three websites.^2^ This sampling method was chosen to mirror the recruitment strategies for the planned online BPT program; i.e., participants in the current study would be similar to those expected to enroll in the online intervention. Parents expressing interest in participating were asked to complete the SNAP-IV, a widely used ADHD rating scale (22, 23), prior to taking part. Those reporting that their child frequently displays 6 or more symptoms of inattention and/or hyperactivity/impulsivity were invited to participate.

Professional participants (educators and healthcare providers) were recruited via existing professional contacts of the researchers, making sure that they represent diverse disciplines and those working in private and public sectors and serving socioeconomically diverse families. No screening procedures were included for professionals wishing to participate.

Prospective participants were sent an online consent form via Whatsapp. Upon completion of the consent form, they were contacted by the researchers to schedule a phone interview. Interviews were conducted from January to June 2021, by a team of three post-doctoral and -masters researchers, under the supervision of a senior psychiatrist (PM). The interviews lasted from 30 min (with professionals) to 1 h (with parents). Interviews were recorded and later transcribed.

Interviews with parents and professionals included questions addressing: (1) experiences of, and barriers to, families accessing psychosocial, in particular behavioral, treatment for ADHD; (2) parents’ level of knowledge about ADHD, comorbid conditions and treatment options (for professionals, this referred to the knowledge of the parents they interact with); and (3) information and support parents want and need to better assist their children and manage their children’s ADHD symptoms. Parent interviews also included questions about the parenting strategies they currently use, difficulties experienced in managing their children’s behavior, challenges experienced during the COVID-19 pandemic, and sources of information they access to learn about ADHD and parenting. Interview questions are presented in the Supplementary material.

An inductive coding strategy was used to explore narratives emerging from the data. Two rounds of coding were carried out using Delve software.^3^ One of the researchers (PB) completed initial coding using a combination of *in vivo* coding (using participants’ own words) and structural coding (codes limited by the topics introduced in the interview questions). Consistent with the goals of the study, the coder looked for words and phrases that indicated (1) experiences of and barriers to families accessing behavioral treatment, (2) information parents currently have, difficulties they and their children experience, and strategies they use, and (3) information and support families need and want, and the preferred modality for accessing them. Given the interviews were conducted during the COVID-19 related restrictions, the researcher separately coded pandemic specific responses, in terms of the difficulties families experienced with their child’s behavior and accessing care. The codes generated through this process were organized into main categories and subcategories. Two additional researchers (RC, CB) subsequently reviewed the data. The three researchers discussed edits and additions to the codes until they reached consensus (see Supplementary material for the categories and main categories identified). Next, the primary coder carried out thematic analysis to identify common themes based on the codes that were frequently referenced in participants’ responses. Research team members (PB, RC, CB, EF) discussed these themes until they reached consensus. There was no criteria as to the minimum number of participants mentioning a theme for the theme to be identified. Rather, efforts were made to create themes that are inclusive of participants’ responses. These themes are presented in narrative form in the Results.

## 3. Results

Table 1 presents participant characteristics. While efforts were made to recruit parents of children receiving healthcare from the public and private sectors, with diverse socioeconomic backgrounds, the participants were mostly middle class and accessed healthcare from the private sector. This likely reflects the fact that the parents learned about the current study online and volunteered to participate; thus they were parents with greater access to resources. Efforts to recruit professional participants in different roles, who work in public and private sectors servicing diverse families, were more successful.

**Table 1:** Participant characteristics.

	Parents		
	(*n* = 23)		
	M	SD	Range
Parent education (years)	16.9	2.9	10–20
Mothers *n*	19 (82%)		
Income (ABEP) class^a^ *n*	A and B1 7 (30%), B2 7 (30%), C1 and C2 9 (40%)		
Services in private sector *n*	Health 18 (78%), education 17 (74%)		
Child’s age (years)	9.3	2.8	4–16
Child with ADHD diagnosis *n*	18 (78%)		
SNAP inattention sum	21.4	3.1	15–27
SNAP hyperactivity/impulsivity sum	18.7	6.3	0–27
	Educators		
	(*n* = 15)		
Professional role *n*	Educational specialist 6 (40%), teacher 5 (33%), school principal 2 (13%), school counselor/psychologist 2 (13%)		
Services in private sector *n*	6 (40%) + 3 (20%) in both		
	Healthcare Providers		
	(*n* = 16)		
Professional role *n*	Psychiatrist 5 (31%), psychologist 5 (31%), speech therapist 3 (19%), pediatrician 3 (19%)		
Services in private sector *n*	7 (44%) + 6 (38%) in both		

a ABEP social economic strata based on average household income estimation (A and B1 > 10, B2: 5–10, C1: 3–5, C2: 1–2 x minimum wage).

Qualitative narratives, generated based on the common themes identified via inductive coding, are presented below. The percentages of participants whose individual responses fit within these themes, and final codes and exemplar responses associated with the themes are presented in the Table 2.

**Table 2:** Codes and exemplar responses associated with the final themes generated from the needs assessment interviews, and the percentage of participants whose individual responses referenced each theme.

	Themes	Participants (%)	Codes	Exemplar responses
Barriers to accessing psychosocial treatment				
*Difficulty obtaining a diagnosis prior to accessing treatment (as identified by parents and professionals)*				
Parent	Takes a long time to receive an appropriate evaluation/diagnosis	74%	Months to see specialists for an evaluation	*“After a pediatrician, took 3 months to see a neurologist who told me to see a psychiatrist, which took 5 months. Still waiting for a psychologist to do a test.”* *“They all had different diagnoses for my son.”*
			Multiple professionals for a diagnosis (with long waits between appointments)	
	Waiting until recommendation/pressure from school to get a diagnosis	57%	Sought a diagnosis after school suggestion	*“I knew my daughter was struggling, but I thought was just the phase. I talked to a doctor after school made me.”* *“It’s hard for me to accept.”* *“I worry that she will not be able to stay in school.”*
			Hesitation due to possible discriminations (if diagnosed)	
Educator	Parents’ difficulty seeking, accepting, or sharing a diagnosis	93%	Long time between notifying a concern and family receiving a diagnosis	*“There are many families that are ashamed.”* *“Some families are slow to accept the child has difficulties.”* *“Families are afraid that children with ADHD will not be able to learn.”*
			Difficulty seeking a diagnosis (financial, motivation, concern over medication)	
			Difficulty accepting child’s difficulties or diagnosis	
			Hesitancy with ‘disorder’ label by families and professionals due to stigma	
			Families hiding a diagnosis	
Healthcare	Parents’ difficulty seeking, receiving, or accepting a diagnosis	35%	Parents unable to identify child’s difficulties	*“Instead of going to a doctor, families look for educational professionals for a diagnosis.”* *“They think that the symptoms will disappear with time and that it is a learning problem.”*
			Parents/schools not seeing ADHD as a clinical disorder	
*A lack of availability of behaviorally oriented treatments, in particular BPT (as identified by parents and professionals)*				
Parent	Has had difficulty receiving any non-pharmacological treatment	52%	No availability of professionals taking health insurance	*“It’s a struggle to find professionals who takes health insurance.”* *“There is a lack of specialists. When I find them, they do not have openings.”*
			A lack of professionals or high-quality services	
			Difficulty getting to treatment due to a lack of transportation (e.g., bus fare)	
	Child has received some type of psychotherapy (vs. not)	43% (57%)	Psychotherapy (incl., CBT, general ‘therapy’, child plays a game with therapist)	*“My child had therapy - he played games and did drawings”* *“The therapist would talk to my child, but not sure what.”*
	Parent involved in child’s treatment (vs. not)	9% (91%)	Meet with a child’s therapist regularly	*“The therapist usually meets with my child but talks to me sometimes.”*
			Received some advice from professionals	
	Parent has received behavior management training (vs. not)	9% (91%)	Any mention of receiving behavior management or parent training	*“Yes, I participated in behavior management training.”*
Educator	Families have difficulties accessing behavioral health care	86%	Parents do not have financial resources to pay for treatment	*“We can convince the families to look for help, sometimes manage to get an appointment at a clinic that accept public health insurance, but then families do not have money for bus ticket to get there.”* *“For many families, getting food is their priority, and they do not think attending to the child’s behavior problem is not that important.”*
			Parents do not recognize the need for treatment (thus do not seek treatment)	
			Lack of appropriate and affordable services	
			Parents/families do not have time to attend treatment	
			Families do not have transportation to attend treatment	
Healthcare	Families have difficulties accessing behavioral health care	100%	Parents do not have financial resources to pay for treatment	*“Specialists do not have appointments available, till many months ahead.”* *“They go to a doctor, who barely evaluates and gives medication that’s free through public insurance - risperidone instead of Ritalin.”* *“Parents go to ‘psychopedagogues’ for help, without knowing they specializes in learning problems.”* *“Many ask about phytotherapies and homeopathy.”* *“Parents do not accept the diagnosis and say, ‘in my time there was no such thing’ [as ADHD].”*
			Have never been offered other types of treatment than medication	
			Parents interested in medication only (due to lack of information on/availability of other interventions)	
			Lack of services in public health care settings	
			Parents do not recognize the need for treatment (thus do not seek treatment)	
			Lack of knowledge about what treatment is appropriate for ADHD	
			Parents/families do not have time to attend treatment	
			Families do not have transportation to attend treatment	
Information parents want/need to better support their children				
*Difficulties children experience (as identified by parents)*				
Parent	ADHD-specific behavior difficulties	96%	Inattention	*“My child lacks focus and forgets what he was doing in the middle of the task.”* *“He runs around and talks a lot - cannot sit still during a meal.”* *“Does things without thinking. When we talk about it he knows what he did wrong, but then does it again.”*
			Hyperactivity	
			Impulsivity	
	Emotional difficulties	43%	Irritability/emotional outbursts	*“My child is very emotional.”* *“He gets hurt very easily.” “He is very insecure.”*
			Anxious/sensitive	
			Cries often	
	Learning/school difficulties	74%	Difficulty completing homework	*“It takes long time to do homework.”* *“My child has problems at school, especially with writing.”*
			Writing difficulties	
	Social difficulties	61%	Shy	*“My child gets angry at her friends with little things, and does not want to play with them anymore.”* *“He is very happy, and sometimes over the top - this drives people away.”*
			Difficulty making friends/getting along with others	
			Difficulty with social communication	
	Non-compliance/needing to repeat directions	30%	Do not follow rules/comply with directions	*“I have to repeat fifty thousand times and he still does not listen.”* *“He never closes the door, never brings his towel to the shower - I told him so many times.”* *“His opposition to rules is very stressful.”*
			Repeat directions over and over	
			Frequent reminders required	
	Struggle with daily routines	65%	Difficulty starting homework	*“We argue about homework all the time - he procrastinates.”*
			Difficulties during meal, bath, and bedtime	
*Difficulties parents/families experience* (*as identified by parents*)				
Parent	Disruption on family relationship	48%	Disturbs other family members’ mood and everyday life	*“My child is loving, but his impulsivity disturbs the peace of the family.”* *“He needs constant attention - wants to talk and show us things all the time.”* *“His brother does not understand him - gets annoyed and fights with him a lot.”* *“I do not have a social life anymore. We cannot even go to church because he will not sit still.”*
			Frequent arguments	
			Constantly require parental attention (parents cannot do other tasks)	
			Cannot go out due to child’s behavior	
	Parents experiencing stress	82%	Emotional burnout/exhaustion/despair	*“I cannot take it anymore.”* *“I’m exhausted.”* *“I sometimes think I’m horrible - it’s not the way I was raised”* *“I have no escape valve at all, it’s very hard.”* *“Nobody takes care of me, it’s hard.”*
			Frustration	
			Insecurity/feeling lost	
			Guilt/frustration about self	
			Feeling sad/crying	
*Information parents want/need to* (*as identified by parents and professionals*)				
Parent	Knowledge about ADHD	52%	When and how ADHD is diagnosed/subtypes are determined (impulsive, inattentive or both)	*“We know that he has ADHD, but do not know exactly what ADHD is and how it is different from a disobedient child.”*
			Which behaviors due to ADHD or something else (lack of interest, stubborn)	
	How to manage child’s behavior	70%	Creating/dealing with everyday routine	*“We want practical information - on how to approach and deal with my child.”* *“I want something simple - like how I can get my child get started on homework.”*
			Dealing with child’s frustration	
			Reducing screen time	
			Help child engage in homework	
	How to manage own behavior/responses to child	60%	Do not know how to act/react to the child	*“When you as a parent lose your limit, what do you do?”* *“I get stressed and angry - do not know what else to do to get my child’s attention.”* *“I’m afraid to praise - it could have negative effects.”*
			Managing own stress	
			Scream less	
			Be more patient	
			How much to praise the child	
	How to deal with other difficulties	78%	Help with learning/academics	*“I do not know how to help my child with his schoolwork.”* *“My biggest concern is that if he does not learn how to deal with these symptoms, he will not be able to take care of himself as an adult.”*
			Child cleaning/organizing their room	
			Communication with school	
			Protecting the child from stigma/discrimination	
			Concerns for future	
Educator	Knowledge about ADHD	80%	How to identify ADHD	*“Parents need to know how to support their child outside school.”* *“Parents need to understand that treatment takes time, and the child will need support throughout much of their school life.”*
			Appropriate treatment	
			Differential diagnosis	
			Long-term consequences	
	How to assist their child	67%	Establishing daily routines	*“Parents need to know how to organize things at home and set up a routine.”* *“Parents need to understand that a child with ADHD needs help, cannot do things alone.”*
			Using positive reinforcement/praise	
			Practical parenting strategies	
			Learning strategies appropriate for the child	
Healthcare	Knowledge about ADHD	70%	Understanding ADHD	*“Families need to really understand what a child with ADHD is like.”* *“We see a lot of ADHD children with parents thinking they are autistic.”* *“Parents are worried about ADHD treatment, because they think medications are addictive, are used at parties and can kill the child.”*
			Differential diagnosis	
			Difference between a clinical disorder and child’s personality, motivation, will.	
			Reduce prejudice about ADHD and other diagnosis	
			Information about medication	
	How to assist their child	47%	Improve daily structure and organization	*“Parents need to learn how to react to their child and how to help the child differently.”* *“Parents need to listen to how the child feels and build trust.”*
			Improve parent–child interaction/communication	
Current parenting strategies employed (as identified by parents)				
Parent	General use of praise	78%	Praise often	*“Oh, I always praise him. I tell him you are wonderful, you are smart and all that.”*
			Tell child ‘I’m proud of you’, ‘good boy’ etc.	
	General use of tangible rewards	17%	Buy things for good behavior	*“I buy him ice cream sometimes.”* *“When he does something well, he asks for a toy, if it’s in my budget, I buy it.”*
	Selective use of positive reinforcement for appropriate behavior	4%	Use ‘positive reinforcement’ for specific behavior	*“It is amazing how well he responds to positive reinforcement.”*
	Use of stimulus/environmental control	13%	Reduce distractions	*“Always try, when he has schoolwork, to take away stimuli, try to put him in a quieter place, away from the door and window.”*
	Use of prompts	8%	Warnings and reminders	*“What I have learned in my daily life, which was a tip from the teacher, is how to give the command.”*
	Organize environment	35%	House rules	*“I tell him to write down things he does not want to forget.”* *“Try to have him use checklists and cell phone alarms.”*
			Checklists	
			Notebook for reminders	
	Talk to the child	56%	Explain consequences	*“Usually, we try to explain what’s right and what’s wrong.”* *“I tell him about consequences, like what happens when he does not do homework.”*
			Explain that they have to do what’s required of them, what’s important	
			Explain how the child’s behaviors make them (parents) feel	
	Negative punishment (take things away)	26%	Take away cell phones, video games	*“Sometimes I threaten him -’if you continue like this, I will take away the cell phone and video game’.”*
	Negative punishment (time out)	13%	Give time out	*“I make her stand still and think for 5 min.”*
	Negative punishment (grounding)	26%	Ground	*“I ask her to reflect and, depending on her behavior, I ground her.”*
	Positive punishment (raised voice)	34%	Yell, scream at child	*“We end up yelling and fighting with him trying to show him that he is wrong.”*
	Positive punishment (physical)	13%	Slap on the hand or in other parts of the body	*“Give a few slaps on the butt.”* *“She cries, she hits, then I slap her too.”*
	General strategies improve mood, family relationship	43%	Try to have fun together as a family	*“We are trying to connect better with him.”*
			Provide care/tenderness	
Difficulties during COVID (as identified by parents)				
Parent	Treatment disruption	30%	Treatment interrupted	*“The therapist stopped treatment in person when the pandemic hit last year.”* *“We are waiting for a psychologist to come back after this pandemic to do the test with him.”*
			Difficulties with online therapy	
			Cannot start treatment	
	Increased difficulties/challenges of child	57%	Behavior regressed	*“He got worse during the pandemic.”* *“He has so much energy from being home and is out of control.”* *“I get stressed seeing him on the computer and cell phone all the time.”*
			Increased screen time	
			Decreased social contacts, extracurricular activities	
	Increased parental stress	70%	Increased child-care responsibility	*“Making him pay attention to online classes is very stressful.”* *“He cannot do online classes on his own, then I cannot do my work.”* *“The pandemic made it difficult for us to get along with each other.”*
			Disruption in daily routines	
			Decreased patience	
Sources and contents of online information accessed (as identified by parents)				
Parent	Sought information online on their own or after recommended by professionals	61%	Professional websites	*“I look for Instagram groups to try to understand my child’s condition.”* *“I looked at the ABDA website and found some articles and books.”* *“I try to look around for information online. I feel that doctors and researchers know a lot, but it’s not being passed on to those who really need it.”*
			Google	
			Instagram	
			YouTube videos	
			WhatsApp parent groups	
	Access online information from computer	52%	Computer	
	Access online information from phone	100%	Phone	
	Difficulties finding practical information online	74%	Difficulty finding online information about practical strategies	*“I follow several websites that gives information about the disorder, but I already know all that. It’s harder to find information that helps me with my child’s everyday behavior.”*

### 3.1. Barriers to accessing psychosocial interventions

Two major barriers to access were identified: (1) difficulty obtaining a diagnosis prior to accessing psychosocial interventions, and (2) a lack of availability of behaviorally oriented treatments, in particular BPT.

Many parents (57%) reported that school personnel initially raised concerns about their child’s behavior, with parents seeking a diagnostic evaluation prior to receiving any treatment. Parents (74%) noted a long delay, and/or needing to see multiple professionals, before obtaining a diagnosis. The reports of educators (93%) and healthcare providers (35%) also reflected delays in children receiving a diagnosis, but also in parents seeking and then accepting their child’s diagnosis. The responses of both parents and professionals were indicative of perceived stigma contributing to the delay in obtaining a diagnosis and subsequent treatment. Healthcare providers noted that parents are often afraid of their child receiving a diagnosis, believing that ADHD is like severe Autism Spectrum Disorder and their child would be excluded from regular education.

The responses of parents (52%) and professionals (86% educators and 100% healthcare providers) also indicated a lack of quality and affordable non-pharmacological services. Among the parents interviewed, while almost half reported their child had received some form of psychotherapy, most indicated no or minimal parental involvement in the treatment (i.e., treatment consisting of a child meeting with a psychologist/psychoanalyst alone). One parent indicated learning behavioral management strategies from professionals involved in the child’s care, and another reported meeting regularly with the child’s psychologist. Two parents reported they received, or were planning to participate in, behavioral management training. Both educators and healthcare providers raised concerns that parents often do not recognize the need for treatment or lack knowledge regarding appropriate treatment for ADHD.

While parents and professionals generally agreed that barriers to treatment access exist and are problematic, parents’ responses focused on the lack of availability while professionals’ responses focused on parents’ reluctance in seeking and accepting support.

### 3.2. Information parents have and want/need to better support their children

The responses of both parents (52%) and professionals (58% educators and 70% healthcare providers) indicated that parents would benefit from having a better understanding of ADHD and its comorbid conditions. In particular, while correctly identifying the symptoms of ADHD and listing behaviors associated with the disorder (96%), parents struggled to identify which of their children’s behaviors are due to ADHD, a comorbid condition, the child’s personality or a lack of motivation. They reported a wide range of difficulties (43% emotional, 74% learning/school, 61% social difficulties, 30% non-compliance, and 65% daily routines). Healthcare providers further reported that many parents believe ADHD is a learning problem and look for a diagnosis and assistance from educational specialists, rather than doctors or psychologists. Educators reported confusion amongst parents regarding their child’s difficulties and needs, also noting some avoidance by healthcare providers to use diagnostic labels.

The majority of parents (70%) referenced the need for information on how to manage their child’s behavior, as well as their own behavior and emotions when interacting with their child (60%). They elaborated that their child’s behavior negatively affected family relationships and that they experienced significant stress. They wanted to know how to make everyday routines easier and more pleasant, with fewer arguments and less frustration. Parents (78%) also noted wanting information on how to assist their child’s learning and organizational difficulties, how to communicate their concerns to their child’s school, and how to prevent their child from experiencing future hardships and being discriminated against. Educators (67%) identified the importance of providing parents with practical strategies on how to assist their child at home. Healthcare providers (47%) reported that families would especially benefit from support to increase structure and to improve communication in the home.

Overall, parents and professionals seemed to agree on the need for parents to have a clearer understanding of factors that contribute to the child’s behavioral difficulties and how to address them. However, even amongst professionals, there may not be consensus as to the specific nature of these difficulties.

### 3.3. Current parenting strategies employed

Parents were also asked about how they currently manage their children’s behavior. One parent was well informed about behavior management techniques consistent with those taught in BPT programs, but others reported having very little knowledge about how to support their children beyond common, generic parenting strategies (e.g., being positive with my child). Many parents reported using rewards, such as praise (78%), food and toys (17%), for good behavior. However, the use of praise was usually non-specific, and some parents questioned the appropriateness or effectiveness of such rewards for behavior management (4% reporting selective use of positive reinforcement for appropriate behavior). Other strategies reported included talking to the child about their behavior (56%) and implementing consequences, e.g., taking things away (26%), and using time out (13%) or grounding (26%). Raising voices (yelling/screaming) was not uncommon (34%), while some reported hitting their child (13%). Many parents noted continued difficulties in managing children’s behavior despite trying multiple strategies.

### 3.4. Difficulties during COVID-19

Interviews took place during the COVID pandemic when most schools were closed. Treatment and evaluation services were also interrupted during this time (30%). Parents reported increased behavioral and emotional difficulties in their children (57%). They also worried about their children spending more time on screen and not having opportunities for social interaction. Many parents (70%) reported increased stress for themselves, due greater demands on them, often juggling work, childcare and assisting with their children’s online schooling.

### 3.5. Online information sources

As we were aware that many parents seek information about children’s behavioral difficulties and parenting strategies online, we included questions about where and how parents seek such information. Parents (61%) reported using popular online platforms such as Instagram and Whatsapp parent groups, as well as accessing professional websites such as ABDA (Brazilian Association for Attention Deficit). All parents (100%) reported accessing such information using their cell phones. While able to access information on the nature of ADHD from these sites, many parents (74%) reported difficulty finding practical information on how to manage their children’s difficulties in everyday life.

## 4. Discussion

A qualitative needs assessment was carried out to understand the experiences and behavioral treatment needs of families with children demonstrating ADHD symptoms. Semi-structured interviews were conducted with parents, educators, and healthcare providers. Common themes were identified via inductive coding of the interview responses. Given these themes are presented in a narrative form in the results, here we highlight those that are most relevant to the study aims.

Many of the parents who volunteered to take part in this study had sought some form of psychosocial treatment, in addition to medication. Among those whose children participated in psychotherapy, the majority reported their child meeting alone with the therapist with little parental involvement in the intervention. A small number of parents reported learning behavioral management strategies from healthcare providers. When parents were probed for the information and support they desired, many reported wanting practical information on how to manage their children’s behaviors in everyday life. They also reported experiencing significant stress and wanting to know how they could better manage their own reactions when their child does not follow their directions. Healthcare providers and educators noted that many families would benefit from learning how to create structure and develop routines in the household, and how to assist and interact with their children with ADHD at home. Some parents and professionals reported that families would also benefit from learning more about ADHD generally. However, many parents reported that information about ADHD can be found online, but that it is more difficult to find information about practical parenting strategies that work with children with ADHD. This highlights the need for demonstrations of such strategies via easily accessible formats, such as short videos.

The need for a formal diagnosis delayed treatment access for many families. This was partly due to the lack of providers offering diagnostic services. However, parents’ hesitancy in seeking and receiving a diagnosis also contributed to this delay. Many parents reported that they only sought out a diagnosis after their child’s school asked them to do so. They noted that it was challenging for them to accept that their child’s difficulties qualified for a diagnosis, or they were worried that having a diagnosis would result in discrimination and loss of educational opportunities. The responses of educators and healthcare providers confirmed that families are reluctant or afraid of seeking a diagnosis. Their responses also indicated that families are not usually aware of treatment options, with many thinking medication is the only choice. Other families prefer homeopathic treatment or educational assistance. Consistent with the literature (2, 3), educators and healthcare providers also described limited availability of accessible non-pharmacological treatment for ADHD.

These data highlight the need for increased availability of accessible non-pharmacological interventions for ADHD. These interventions should have empirical support to reduce commonly reported behavioral difficulties of children with ADHD and parental distress. Behavioral parent training fits this criteria. A pre-diagnostic, and/or post diagnosis, early behavioral intervention might be appropriate for Brazilian communities. Behavior management strategies can be useful for parents who have concerns about their child’s behavior, but have not sought or received a diagnosis for the child. Such early intervention programs have been disseminated successfully in other countries (24–26); for example, specific treatment recommendations, or treatment itself, are provided at regular developmental check-ups and through schools. While a careful diagnostic evaluation is important in developing individually tailored treatment plans, it is also important to reduce delays in families accessing accurate information about ADHD and behavioral strategies to help manage children’s behavior.

Ease of access is critical in the uptake of such early intervention programs. One way to disseminate behavioral management strategies for free or at low cost to families may be via online platforms. Such an approach is foreshadowed in calls for tiered child mental health care involving digital tools in Brazil (27). BPT programs have been offered online in other countries with emerging empirical support (28–30). Many Brazilians access information online with cell phones, which are widely available even amongst low-income communities (31, 32). Phone-based digital platforms could be considered for dissemination of behavioral parent training for families of children with mild to moderate ADHD.

The current study provided an opportunity to hear directly from parents, and those working with families, what is needed to better support families of children with ADHD in Brazil, albeit with a relatively small sample. In recruiting parents, we relied on their reports of their children’s symptoms. Thus, the sample includes those with a formal diagnosis of ADHD as well as those demonstrating elevated symptoms of ADHD, increasing the generalizability of the findings. The parents learned about the current study online and volunteered to participate; thus they were likely a sample of motivated parents with resources to access the study information and are not representative of the entire Brazilian population needing treatment for their children with ADHD. While this sampling method mirrored the recruitment strategy planned for the online program in development, this may have impacted the findings. Among the lower-resourced families, the availability of affordable treatment is likely even more scarce, and the acceptance and knowledge about behavioral disorders and treatment is likely more limited (33, 34). As a counterpoint, responses from educators and healthcare providers working in both private and public sectors provided diverse perspectives. As in all qualitative research, the influence of the researchers’ viewpoints and experiences needs to be acknowledged. However, we used consensus among three researchers from different professional backgrounds (psychiatrist, psychologist, neuroscientist) in coding the data, which were then reviewed by two senior researchers (GT, PM) who were not involved in data collection.

The in-depth interviews with the stakeholders of a planned online BPT program provided important insights regarding necessary content and possible delivery strategies. The themes emerging from the current study, and the literature on psychosocial treatment for ADHD, also suggest behavioral parent training programs would be an important addition to child mental health services in Brazil. Such programs should be easily accessible, offer practical strategies for dealing with everyday life challenges, and provide support for parents. The dissemination of such programs would help address existing treatment gaps for Brazilian children with ADHD and their families. To support such dissemination, community wide educational programing may also be needed. Helping parents, educators, and healthcare providers understand the importance of recognizing ADHD and receiving caregiver-focused therapy would increase the acceptability of parent training programs. Given that school personnel are usually the first to raise concerns about the child’s behaviors and act as facilitators for help seeking, schools may be particularly suited for such educational programming and for reaching the families who may benefit from an online BPT program.

## Data availability statement

The raw data supporting the conclusions of this article will be made available by the authors, without undue reservation.

## Ethics statement

This study involving human participants was reviewed and approved by the D’Or Institute for Research and Education Ethics Committee (CEP #5249). The participants provided their written informed consent to participate in this study.

## Author contributions

GT, PM, and EF contributed to the conception and design of the study. PB, RQ, and CB organized the database and performed the qualitative analysis. PB and EF wrote the first draft of the manuscript. All authors contributed to the manuscript revision, read, and approved the submitted version.

## Funding

This study was supported by a joint research agreement between Okinawa Institute of Science and Technology (OIST) and D’Or Institute for Research and Education (IDOR).

## Conflict of interest

PM has received research grant and speaker honoraria from Takeda in the last 3 years.

The remaining authors declare that the research was conducted in the absence of any commercial or financial relationships that could be construed as a potential conflict of interest.

## Publisher’s note

All claims expressed in this article are solely those of the authors and do not necessarily represent those of their affiliated organizations, or those of the publisher, the editors and the reviewers. Any product that may be evaluated in this article, or claim that may be made by its manufacturer, is not guaranteed or endorsed by the publisher.

## Footnotes

^1^ https://www.nice.org.uk/guidance/ng87/chapter/Recommendations#managing-adhd

^2^www.habitepais.com.br; https://www.rededorsaoluiz.com.br/instituto/idor; https://tdah.org.br/

^3^ https://delvetool.com/

## Supplementary material

SUPPLEMENTARY DATA SHEET 1 Interview questions.

SUPPLEMENTARY DATA SHEET 2 English translation of interview questions.

SUPPLEMENTARY DATA SHEET 3 Categories and subcategories for initial coding.
