# Supplementary material for: Needs assessment for behavioral parent training for ADHD in Brazil
Source: Front Psychiatry. 2023 Jul 27;14:1191289. doi: 10.3389/fpsyt.2023.1191289 (PMC10415012; doi:10.3389/fpsyt.2023.1191289)
Supplement: SUPPLEMENTARY DATA SHEET 1 — Interview questions. [file Data_Sheet_1.DOCX]

**Avaliação de necessidades: Perguntas para entrevista com os pais**

**Identificação do participante:**

**Informações demográficas**

A1. Cidade/bairro:

A2. Renda familiar:

☐ < 1 salário mínimo

☐ Entre 1 e 2 salários mínimos

☐ Entre 3 e 5 salários mínimos

☐ Entre 5 e 10 salários mínimos

☐ > 10 salários mínimos

***Criança com TDAH e problemas relacionados***

A3. Idade:

A4. Sexo: ☐Feminino ☐ Masculino

A5. Tipo de escola: ☐Pública ☐Particular

A6. Tipo de busca a tratamentos (público vs privado): ☐Público ☐ Privado

A7. Número de crianças em casa:

***Informações sobre o cuidador primário***

A8. Relação com a criança: ☐Mãe ☐Pai ☐Avó ☐Avô ☐Outro:

A9. Grau de escolaridade (Ensino médio, superior completo, pós graduação)::

A10. Ocupação atual:

A11. Já teve diagnóstico ou trata de alguma condição relacionada à sua saúde mental? (Ansiedade, depressão, etc) Qual?

A12.Você se considera a pessoa mais envolvida no cuidado com a criança? ☐Sim ☐Não

A13. Outros adultos envolvidos no cuidado da criança:

☐Avó ☐Avô ☐ Tia ☐Tio ☐Irmãos ☐Babá/Empregada ☐Outros:

**Discurso Livre:**

1. Me fale como é o comportamento de seu(a) filho(a) no dia-a-dia
2. Como está o relacionamento de vocês ou o convívio familiar?

**Dificuldades da criança**

1. Seu(a) filho(a) foi diagnosticado com TDAH, certo? Como ele recebeu esse diagnóstico?
2. Além do TDAH, seu filho(a) tem algum outro diagnóstico formal, dificuldades de comportamento ou de aprendizado?

☐Dislexia

☐Discalculia

☐TOD (T. desafiador opositivo)

☐Transtorno de Conduta

☐Transtorno de Desregulação do humor

☐Autismo/Asperger

☐Depressão/Ansiedade

☐Outros

1. O que você sabe sobre os sintomas de TDAH?
2. O que você sabe sobre como lidar com os sintomas do TDAH?
3. O que você sabe sobre sintomas de XXXX (resposta da pergunta 4) em crianças? Sabe como lidar com isso?
4. Qual a sua maior preocupação em relação à criança? (ex.: comportamento opositivo, aprendizado, habilidades sociais)
5. O que você faz geralmente quando seu(a) filho(a) se comporta mal ou age de alguma maneira que você não gosta/concorda?
6. Como ele(a) reage quando você faz isso?
7. E o que você faz quando seu(a) filho(a) tem um bom comportamento ou age de acordo com o que você gostaria?

**Desafios/Dificuldades dos pais**

1. Quais as principais dificuldades que você, como mãe/pai tem vivenciado? O que você considera mais estressante?
2. O que você gostaria de poder mudar na sua relação com seu(a) filho(a)?

**Acesso ao tratamento**

1. Seu filho já recebeu algum tratamento ou ajuda?

☐Médico/Farmacológico

☐Psicoterapia

☐Pedagogia

☐Fonoaudiologia

☐Aulas particulares ou de reforço

☐Outros:

**Se não houver recebido nenhum tratamento, pular para a pergunta número 20:**

1. Se a criança recebeu tratamento profissional **não-farmacológico**: qual tipo de tratamento seu filho recebeu? (ex: terapia cognitiva, treinamento de pais)?
2. Você observou resultados positivos?
3. O que tornaria esse tratamento melhor? (ex. mais barato, maior facilidade no acesso ao tratamento)?
4. Se a criança recebeu tratamento profissional não-farmacológico, (ex: terapia cognitiva, treinamento de pais), como você conseguiu esse tratamento? Foi recomendado a você por algum profissional ou pela escola?

☐Recomendado por um profissional de saúde

☐Recomendado pela escola/professores

☐Busquei por conta própria

1. Foi fácil conseguir este tratamento? Você foi orientado em algum momento sobre qual tratamento seria melhor e onde poderia procurar esse serviço?

_____________________________________________________________________________________

**Se a criança estiver recebendo algum tratamento, não preencher as perguntas 20 e 21**

1. Quais são as razões de **não receber o tratamento**? (ex. não ser necessário, medicação ser suficiente, dificuldade em encontrar um terapeuta, custo financeiro do tratamento, tempo de transporte até o serviço)
2. Se existisse um terapeuta disponível para você, com baixo custo, você gostaria de receber tratamento não-farmacológico? ☐ Sim ☐Não ☐Talvez ☐Não sei
3. Além de um tratamento profissional (medicamentoso ou não-medicamentoso) você procurou alguma outra fonte de informação ou apoio? Alguma coisa foi recomendada a você? (ex., tratamento medicamentoso, grupo de apoio, sites, livros, acomodações escolares)
4. Eles trouxeram resultados positivos/ajudaram? O que você achou que foi útil e o que não foi?
5. Que tipo de informação você gostaria de ter? (ex.: sobre o transtorno, como lidar com algum comportamento, grupo de apoio, adaptações escolares)

**Uso da internet**

1. Como você costuma acessar informações online?

☐Celular

☐Computador

☐Outro:

1. Você costuma mandar mensagens? Qual aplicativo você costuma usar para mandar mensagens? (ex., Whatsapp)

☐Whatsapp

☐Telegram

☐Facebook

☐Messenger

☐Outros:

1. Você assiste ou já assistiu vídeos online? ☐Sim ☐ Não
2. Onde você costuma assistir vídeos?

☐No celular

☐No computador

☐Outros:

29. O que geralmente determina se você usa ou não um aplicativo ou se segue alguma página na internet?

30. Durante a pandemia do COVID-19, alguns profissionais estão oferecendo tratamento online. Esse acesso online tornou o tratamento mais fácil ou mais difícil pra você?

☐Mais fácil

☐Mais difícil

☐Não fez diferença

☐Não se aplica (não buscou/ não modificou)

**Projeto Intervenção**

31. Estamos planejando desenvolver um programa online que forneça informações para pais com o objetivo de ajudar o manejo com crianças com TDAH e outras dificuldades relacionadas. Planejamos utilizar vídeos curtos. Você gostaria de receber informações sobre esse programa durante o desenvolvimento dele e nos dar opiniões e sugestões sobre ele?

☐Sim ☐Não ☐Talvez
