# Supplementary material for: Needs assessment for behavioral parent training for ADHD in Brazil
Source: Front Psychiatry. 2023 Jul 27;14:1191289. doi: 10.3389/fpsyt.2023.1191289 (PMC10415012; doi:10.3389/fpsyt.2023.1191289)
Supplement: SUPPLEMENTARY DATA SHEET 2 — English translation of interview questions. [file Data_Sheet_2.docx]

**Needs Assessment: Parent Interview Questions (English translation)**

**Participant identification:**

**Demographic information**

A1. City/District:

A2. Family income:

☐ < 1 minimum wage

☐ Between 1 and 2 minimum wages

☐ Between 3 and 5 minimum wages

☐ Between 5 and 10 minimum wage

☐ > 10 minimum wages

***Child with ADHD and related issues***

A3. Age:

A4. Sex: ☐Female ☐ Male

A5. Type of school ☐Public ☐Private

A6. Treatment Search type (public vs private): ☐Public ☐ Private

A7. Number of children at home

***Primary caregiver information:***

A8. Relationship with the child: ☐Mother ☐Father ☐Grandmother ☐Grandfather ☐Other:

A9. Education level (High school, Higher education, post graduation):

A10. Current occupation:

A11. Have you ever been diagnosed or treated for any condition related to mental health? (Anxiety, depression, etc.). Which one?

A12. Do you consider yourself as the primary caregiver for the child? ☐Yes ☐No

A13. Other adults involved in child’s care:

☐Grandmother ☐Grandfather ☐ Aunt ☐Uncle ☐Brothers ☐Nanny/Maid ☐Other:

**Open-ended questions:**

1. Tell me about your child’s behavior day-to-day.
2. How is the child relationship with you and your family?

**Child difficulties**

1. Your child has been diagnosed with ADHD, right? How did he receive the diagnosis?
2. In addition to ADHD, does your child have any other formal diagnosis, behavior difficulties or learning difficulties?

☐Dyslexia

☐Dyscalculia

☐ODD (Oppositional Defiant Disorder)

☐Conduct Disorder

☐Mood Dysregulation Disorder

☐Autism Spectrum Disorder

☐Depression/Anxiety

☐Other

1. What do you know about ADHD symptoms?
2. What do you know about dealing with ADHD symptoms?
3. What do you know about XXXX (answer from question 4) symptoms in children? Do you know how to deal with it?
4. What is your biggest concern regarding the child? (e.g., oppositional behavior, learning, social skills)
5. What do you usually do when your child misbehaves or acts in ways you don’t like/approve?
6. How does he/she react when you do this?
7. And what do you do when your child behaves well or in the way you like/approve?

**Parent’s Challenges/Difficulties**

1. What are the main difficulties you, as a mother/father, you experience? What do you find most stressful?
2. What would you like to change in your relationship with your children?

**Access to treatment**

1. Has your child ever received any treatment or help?

☐Medical/Pharmacological

☐Psychotherapy

☐Pedagogy

☐Speech Therapy

☐Private lessons or tutoring

☐Others:

**If no treatment has been received, skip to question number 20:**

1. If the child received non-pharmacological treatment: What type of treatment did your child receive? (e.g., cognitive therapy, parent training)?
2. Have you noticed positive results?
3. How can treatment be improved? (e.g., cheaper, easier access to treatment)
4. If the child received non-pharmacological treatment (e.g., cognitive therapy, parenting training), how did you get to this treatment? Did you find it yourself? Recommended by a professional or school?

☐Recommended by healthy professional

☐Recommended by school/teacher

☐I searched on my own

1. Was it easy to access to this treatment? Did you receive any support as to treatment choices and how to seek services?

_____________________________________________________________________________________

**If the child is receiving any treatment, do not complete questions 20 and 21**

1. What are the reasons for not receiving treatment? (e.g. not necessary, medication is sufficient, difficulty finding a therapist, financial cost, transport time to the service)
2. If there was a therapist available to you at a low cost, would you want to receive a non-pharmacological treatment?

☐ Yes ☐No ☐Maybe ☐I don’t know

1. Besides professional treatment (pharmacological or non-pharmacological), did you search for another source of information or support? Other things recommended to you? (e.g., pharmacological treatment, support group, websites, books, school accommodation)
2. Did they lead to positive results? What did you find them useful/helpful?
3. What kind of information would you like to receive? (e.g., about the disorder, how to handle a behavior, support group, academic accommodation)

**Internet Use**

1. How do you usually access online information?

☐ Cellphone

☐ Computer

☐ Other

1. Do you usually text/send messages? What app do you usually use to send texts/messages? (e.g., Whatsapp)

☐Whatsapp

☐Telegram

☐Facebook

☐Messenger

☐Other:

1. Do you watch or had watched online videos? ☐Yes ☐ No
2. Where do you usually watch videos?

☐ On the cellphone

☐ On the computer

☐ Other

29. What usually determines whether or not you use an app or follow a page on internet?

30. During the COVID-19 pandemic, some professionals are offering treatment online. Did this online access make the treatment easier or more difficult for you?

☐Easier

☐Harder

☐No difference

☐Does not apply

**Intervention Project**

31. We are planning to develop an online program that will provide information for parents to help manage children with ADHD and other related difficulties. We plan to use short videos. Would you like to receive information about this program during its development and give us your thoughts and suggestions about it?

☐Yes ☐No ☐Maybe
