# Supplementary material for: Needs assessment for behavioral parent training for ADHD in Brazil
Source: Front Psychiatry. 2023 Jul 27;14:1191289. doi: 10.3389/fpsyt.2023.1191289 (PMC10415012; doi:10.3389/fpsyt.2023.1191289)
Supplement: SUPPLEMENTARY DATA SHEET 3 — Categories and subcategories for initial coding. [file Data_Sheet_3.DOCX]

**Categories**

*Subcategories*

*Portuguese words related to each subcategories were used for coding.

**Parents**

**Child difficulties**

*ADHD-related behavior*

*Inattention*

*Hyperactivity*

*Impulsivity*

*Oppositional behavior/non-compliance*

*Social difficulties*

*Mood/emotional difficulties*

*Daily difficulties*

*Timing/organizational difficulties*

*School difficulties*

*Motivation difficulties*

**Child’s reactions to being disciplined**

*Emotional reactions*

*Awareness about one’s behavior*

*Unable to change behavior*

*Oppositional/defiant behavior*

**Parenting strategies currently used**

*Punishments*

*Rewards*

*Talking*

*Structures*

**Family’s difficulties**

*Child not listening to parents*

*Active disobedience/non-compliance*

*Daily activities*

*Parent’s difficulty meeting the child’s needs*

*Disrupted family relationship*

*Reduced quality of life*

*Helping with schoolwork*

**Parent difficulties**

*Emotional reactions*

*Personal time/life*

**Pandemic-related struggles**

*Child’s difficulties*

*Parent difficulties*

**Difficulty receiving diagnoses**

*Experience of getting a diagnosis by healthcare professionals*

*Parent recognition of child’s difficulties*

*Referral process*

*Testing*

*Parent feelings about diagnoses*

**Difficulty receiving treatment**

*Lack of support*

*Lack of non-medical treatment*

*Lack of professionals*

*Lack of professionals taking health-insurance*

*Logistic difficulties*

*Professional quality/specialty*

**Parent concerns/training needs**

*Dealing with daily difficulties*

*Dealing with ADHD-related difficulties*

*Dealing with emotional reactions and non-compliance*

*Identifying reasons for child’s difficulties*

*Dealing with school difficulties*

*Not knowing how to react to the child*

*Parental stress*

*Information about ADHD*

**Concerns with ADHD diagnosis**

*Stigma*

*Discrimination*

*Education*

*Future*

**Sources of information**

*Books/articles*

*Professional websites*

*Social media*

*Google search*

*Professional services*

*Parent groups*

*Access via computer*

*Access via cellphone*

*In-person*

**Educators**

**Families’ difficulties dealing with ADHD**

*Family disorganization*

*Stigma, shame, fear*

*Misunderstanding*

*Difficulties related to the diagnosis/diagnostic label*

**Families’ difficulties receiving treatment/accommodation**

*Logistical/Financial difficulties*

*Lack of motivation*

*Lack of trust in professionals*

*Getting proper diagnoses prior to treatment*

*Delay in getting diagnoses or treatment*

*Lack of knowledge about treatment choice*

**ADHD-related difficulties observed (at school)**

*ADHD-related behavior*

*Inattention*

*Hyperactivity*

*Impulsivity*

*Oppositional behavior/non-compliance*

*Aggression*

*Social difficulties*

*Mood/emotional difficulties*

*Executive functioning difficulties*

*Learning difficulties*

*Comorbid conditions*

**Families’ perceived needs**

*Organization/routines*

*Awareness of/attitudes toward the child’s difficulties*

*Identifying/understanding ADHD-related difficulties*

*Daily difficulties*

*Parenting strategies*

*Stigma*

*Communication with school*

*Use of positive reinforcement*

**Healthcare providers**

**ADHD-related difficulties reported**

*ADHD-related behavior*

*Inattention*

*Hyperactivity*

*Impulsivity*

*Comorbid psychiatric/neurological disorders*

*Learning difficulties*

*Family conflicts*

*‘Personality’ issues*

*Family pathology*

**Families’ understanding of ADHD**

*Self-diagnosis/diagnoses by non-healthcare professionals*

*Misunderstanding about ADHD*

*Misunderstanding about ADHD treatment*

*Differentiating ADHD-related and other difficulties*

**Families’ difficulties receiving treatment**

*Problems with public health services*

*Difficulties using health insurance*

*Lack of compete diagnostic evaluations*

*Professionals’ awareness about treatment choices*

*Parents ‘awareness about treatment choices*

*Lack of professionals providing non-pharmacological treatment*

*Lack of professionals providing evidence-based treatment*

*Logistic/financial difficulties*

*Confusions about treatment due to comorbid conditions*

*Lack of motivation/persistence*

*Driven by demands from school*

*Mistrust in medical professionals*

*Preference for non-medical services*

*Preference for medication*

*Stigma*

*Socioeconomical and cultural impacts*

**Families’ perceived needs**

*Organization/structures/routines*

*Family conflicts*

*Inconsistency among caregivers*

*Caregiver availability*

*Family communication*

*Psychoeducation*

*Parenting strategies*

*Care appropriate for the nature/severity of the child’s difficulties*

*Socialization*

*Support for receiving appropriate services*

*Mediation with school*

*Understanding treatment choices*

*Use of positive reinforcement*

*Avoiding punishment*

*Understanding medication*

*Understanding non-child focused intervention*

*Stigma*
